# Metabolic Shifts Induced by Fatty Acid Synthase Inhibitor Orlistat in Non-small Cell Lung Carcinoma Cells Provide Novel Pharmacodynamic Biomarkers for Positron Emission Tomography and Magnetic Resonance Spectroscopy

**DOI:** 10.1007/s11307-012-0587-6

**Published:** 2012-08-11

**Authors:** Madhuri Sankaranarayanapillai, Nianxiang Zhang, Keith A. Baggerly, Juri G. Gelovani

**Affiliations:** 1 Department of Experimental Diagnostic Imaging, The University of Texas M. D. Anderson Cancer Center, 1515 Holcombe Blvd, Houston, TX 77030 USA; 2 Department of Bioinformatics and Computational Biology, The University of Texas M. D. Anderson Cancer Center, 1515 Holcombe Blvd, Houston, TX 77030 USA

**Keywords:** FASN, Orlistat, Biomarkers, MRS, Metabolic changes

## Abstract

**Purpose:**

Abnormal fatty acid (FA) synthesis is one of the common features of cancer. Fatty acid synthase (FASN), a multifunctional enzyme playing a key role in biosynthesis of FA, is up-regulated in prostate, breast, and lung carcinomas. Orlistat is a FDA-approved anti-obesity drug that inhibits the thioesterase domain of FASN, interferes with cellular FA synthesis, can arrest tumor cell proliferation, and induces tumor cell apoptosis. The current study was aimed to investigate the metabolic changes associated with FASN inhibition by orlistat and to understand the molecular mechanisms behind the observed metabolic changes in non-small cell lung carcinoma (NSCLC) cell lines.

**Procedures:**

Changes in metabolite pools in four NSCLC cell lines (H441, H1975, H3255, and PC14) with different mutational profiles were studied using NMR spectroscopy before and after *in vitro* incubation with sub-toxic concentration of orlistat and [1-^13^C]d-glucose or [1,2-^13^C_2_]choline. *In vitro* radiotracer accumulation assays in cells were performed with [^3^H]acetate, [^14^C]fluoroacetate, and 2-deoxy-2-[^18^F]fluoro-d-glucose. In parallel, microarray profiling of genes involved in the regulation of carbohydrate and lipid metabolism was performed.

**Results:**

In orlistat-treated NSCLC cells, FASN inhibition results in characteristic changes in intermediary metabolites (FAs, choline, phospholipids, and TCA cycle metabolites) as observed by magnetic resonance spectroscopy. Further, FASN inhibition by orlistat induces multiple adaptive changes in FA synthetic pathway and associated metabolic pathways, including induction of ketone metabolism and glutaminolysis, as well as the up-regulation of 5' adenosine monophosphate-activated protein kinase.

**Conclusions:**

These observed changes in metabolic pools in orlistat-treated cells demonstrate the critical role of fatty acid *de novo* synthesis and metabolism for cellular energy production, especially in tumor cells with low glycolytic activity, which goes beyond the widely accepted concept that FA synthesis is important for cell membrane biosynthesis in rapidly proliferating tumor cells.

**Electronic supplementary material:**

The online version of this article (doi:10.1007/s11307-012-0587-6) contains supplementary material, which is available to authorized users.

## Introduction

Overexpression of fatty acid synthase (EC 2.3.1.85, FASN) and increased magnitude of fatty acid (FA) synthesis are common characteristics of many malignancies, particularly those with poor prognosis [1]. The constitutive over-expression of FASN in tumor cells is largely due to dysregulation of signal transduction mechanisms that down-regulate FASN expression in normal cells [2]. Numerous studies have demonstrated high levels of FASN expression in various malignant and pre-malignant lesions [3, 4], including lung carcinomas [5].

Pharmacologic inhibition of FASN by small molecule inhibitors or down-regulation of FASN gene expression by RNAi results in apoptosis of cancer cells, inhibition of tumor growth, and prolongation of survival of tumor-bearing animals [2]. Several inhibitors of FASN catalytic activity have been developed as chemotherapeutic agents. Cerulenin and C75, which target the ketoacyl synthase domain of FASN, were reported as effective small molecular inhibitors of FASN activity in tumor cells [6]. Orlistat is a β-lactone-containing inhibitor of the thioesterase domain of FASN. Inhibition of FASN by C75 and orlistat induces cell death in a variety of tumor cell lines and effectively inhibits the growth of prostate and breast carcinoma xenografts in mice [3, 7, 8]. In contrast, a high level of FAs in the diet (i.e., due to supplementation of omega-3 FAs or fish oil) increases the incidence of cancer in mice with inflammatory bowel disease [9]. Together these data confirm the important role of FASN in cancer development, maintenance, and progression.

Although extensive research has been carried out to investigate the potential of FASN as a promising therapeutic target for cancer treatment, limited studies are available on the metabolic consequences of FASN inhibition in cancer cells. In a previous study, magnetic resonance spectroscopy (MRS) was used to monitor FASN inhibition by orlistat, and phosphocholine (PC) was identified as a potential metabolic biomarker of FASN inhibition [10]. The current study was aimed to investigate the metabolic changes associated with FASN inhibition in non-small cell lung cancer (NSCLC) cell lines with various molecular genetic abnormalities that affect FASN expression activity levels. Changes in the metabolic profiles of the NSCLC cell lines in response to orlistat treatment were studied using multinuclear MRS, which expanded previously reported observations [10]. Further, to identify the molecular mechanisms behind the observed metabolic changes determined using MRS in orlistat-treated NSCLC cells, we used real-time polymerase chain reaction (PCR) arrays to assess changes in the magnitude of expression of various genes involved in the regulation of cellular metabolism. The results of this study demonstrated that FASN inhibition by orlistat triggered several adaptive compensatory changes in several metabolic pathways that could be used as pharmacodynamic biomarkers of FASN inhibition for MRS and PET imaging.

## Materials and Methods

### Cell Lines and Culture Conditions

Human NSCLC cells H441, H1975, and H3255, PC14 were obtained from American Type Culture Collection (ATCC, VA, USA). The mutational characteristics of these cell lines are listed in Table S1 in the “Electronic Supplementary Material”. Cells were routinely cultured in DMEM/F-12 (Invitrogen) supplemented with 5 % FBS (Hyclone, Logan, UT) and 10,000 units/ml penicillin, 10,000 μg/ml streptomycin, and 25 μg/ml amphotericin B (Life Technologies) at 37 °C in 5 % CO_2_.

### Cytotoxicity Assay

The viability of cells in culture following incubation for 24 h with different concentrations of orlistat was assessed using WST-1 assay (Roche, Indianapolis, IN, USA) according to the manufacturer’s protocol. Additional details are provided in the “Electronic Supplementary Material”.

### FASN Activity Assay

Following incubation of tumor cells with 30 μM orlistat or DMSO for 24 h, protein extracts were obtained under non-denaturing conditions. FASN activity was determined as described in the “Electronic Supplementary Material”.

### *In Vitro* Radiotracer Accumulation Studies

The rates of accumulation of 2-deoxy-2-[^18^F]fluoro-d-glucose ([^18^F]FDG), [^3^H]acetate, and [^14^C]fluoroacetate in different NSCLC cells were determined using a triple-label *in vitro* radiotracer accumulation assay as previously described [11]. Additional details are provided in the “Electronic Supplementary Material”.

### MRS Studies

For ^13^C labeling of glucose and choline, d-glucose (8.76 mM) in the medium was replaced by equal concentrations of [1-^13^C]d-glucose (Cambridge Isotopes, MA, USA) and unlabeled d-glucose and choline chloride in the medium was replaced by [1,2-^13^C_2_]choline chloride (Cambridge Isotopes, MA, USA) (64.1 μM). H441, H1975, H3255, and PC14 cells were treated with medium containing [1-^13^C]d-glucose and [1,2-^13^C_2_]choline in the presence of 30 μM orlistat or DMSO for 24 h. Cells (~3 × 10^7^–4 × 10^7^) were then extracted using a dual-phase method as described previously [12]. Changes in concentrations of individual metabolites in orlistat-treated cells were expressed as %control. Additional details are provided in the“Electronic Supplementary Material”.

### Gene Expression Analyses

The RNA was isolated from NSCLC cells and purified using RNAeasy kit (Qiagen) and reverse-transcribed to cDNA using the RT² First Strand cDNA Kit (SABiosciences, MD, USA). The expression levels of 168 key genes involved in different metabolic pathways related to FA synthesis and metabolism were determined using custom-designed RT^2^ profiler PCR arrays (SABiosciences, MD, USA) according to the manufacturer’s protocol. The fold changes in gene expression levels were calculated with ΔΔCt method using data analysis software provided by the manufacturer (SABiosciences, MD, USA). Additional details are provided in the “Electronic Supplementary Material”.

### Statistical Analyses

The difference between control and orlistat-treated groups was assessed using an unpaired two-tailed Student’s *t*-test, with differences considered significant for *p <* 0.05. Hierarchical clustering with one Pearson correlation metric and complete linkage rule were used for comparing the gene expression profiles of different NSCLC cells following orlistat treatment.

Pearson correlation was used to determine the correlation between rates of radiotracer accumulation and metabolic changes as determined by MRS and gene expression profiles.

## Results

### Effect of Orlistat on Cell Viability

H441, H1975, H3255, and PC14 cells were treated with different concentrations of orlistat ranging from 25 to 125 μM, and the effect of orlistat on cell viability was determined by WST-1 assay as described. In all the cell lines studied, treatment with orlistat did not have a significant effect on cell viability up to a concentration of 125 μM. A concentration of 30 μM orlistat was selected for subsequent studies, as it has been demonstrated that this dose reduced cellular FA synthesis by ~75 % within 30 min, as determined by the incorporation of [^14^C]acetate into FAs [13].

### Inhibition of FASN Activity by Orlistat in NSCLC Cells

**Fig. 1:**
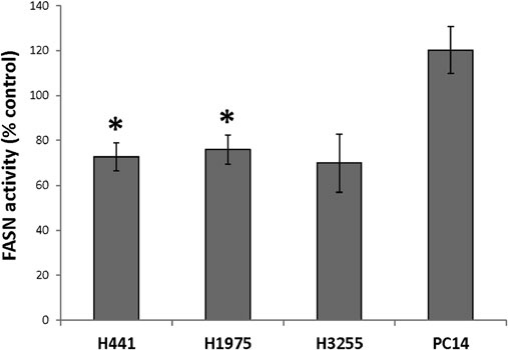
Changes in enzymatic activity of FASN in orlistat-treated NSCLC cells as compared to control (*p < 0.05).

Treatment with 30 μM orlistat for 24 h resulted in a significant inhibition of FASN activity in H441 and H1975 cell lines. FASN activity decreased to 73 ± 6 % in H441 and 76 ± 7 % in H1975 cells (*p* < 0.05), respectively. Orlistat treatment inhibited FASN activity in H3255 cells but did not reach statistical significance (*p* = 0.06). However, in PC14 cells, orlistat treatment did not significantly affect FASN activity (Fig. 1).

### *In Vitro* Accumulation of [^3^H]Acetate, [^14^C]Fluoroacetate, and [^18^F]FDG

In all the cell lines studied, the rate of accumulation of [^3^H]acetate was the highest, followed by [^14^C]fluoroacetate, whereas [^18^F]FDG was found to be the least accumulated radiotracer (Fig. S1 in the “Electronic Supplementary Material”). In particular, H3255 cells demonstrated the highest accumulation rates of [^3^H]acetate and [^14^C]fluoroacetate and the lowest accumulation rate of [^18^F]FDG (Fig. S1c in the “Electronic Supplementary Material”).

**Fig. 2:**
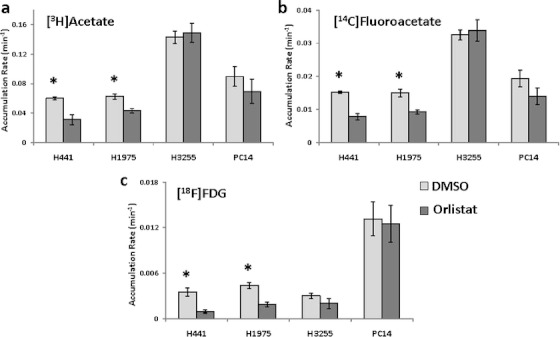
Comparison of unidirectional influx rates *Ki* of **a** [^3^H]acetate, **b** [^14^C]fluoroacetate, and **c** [^18^F]FDG in control (DMSO) and orlistat-treated NSCLC cells (*p < 0.05).

When the cells were treated with 30 μM orlistat for 24 h, the accumulation rates of [^3^H]acetate, [^14^C]fluoroacetate, and [^18^F]FDG decreased significantly in H441 and H1975 cells, as compared to control (*p* < 0.05). However, in H3255 and PC14 cells, orlistat treatment did not significantly affect the accumulation rate of [^3^H]acetate, [^14^C]fluoroacetate, and [^18^F]FDG (Fig. S1 in the “Electronic Supplementary Material”; Fig. 2).

To assess the magnitude of orlistat-induced changes in the *de novo* FA synthesis vs. glycolysis, the accumulation rates of [^3^H]acetate and [^14^C]fluoroacetate were normalized by that of [^18^F]FDG (Table S2 in the “Electronic Supplementary Material”). The ratios of accumulation rates of [^3^H]acetate versus [^18^F]FDG and [^14^C]fluoroacetate versus [^18^F]FDG were relatively higher in H3255 cells, but lower in PC14, as compared to other cell lines. Following orlistat treatment, the relative accumulation of [^3^H]acetate versus [^18^F]FDG and [^14^C]fluoroacetate versus [^18^F]FDG increased significantly in H441, H1975, and PC14. However, in orlistat-treated cells, the ratio between the rates of accumulation of [^3^H]acetate and [^14^C]fluoroacetate did not change significantly, except for H1975 (Table S2 in the “Electronic Supplementary Material”).

### Changes in Metabolite Pools After Treatment with Orlistat

To study the effect of orlistat-induced inhibition of FASN on different metabolic pathways, such as glycolysis, FA synthesis, and choline metabolism, the cells were labeled with [1-^13^C]d-glucose and [1,2-^13^C_2_]choline or [2-^13^C]acetate, as described earlier. ^13^C MRS was performed in control and orlistat-treated cells to assess changes in *de novo* synthesis of various intermediary metabolites. ^1^H and ^31^P MRS were performed to determine changes in the total pool of metabolites.

**Fig. 3:**
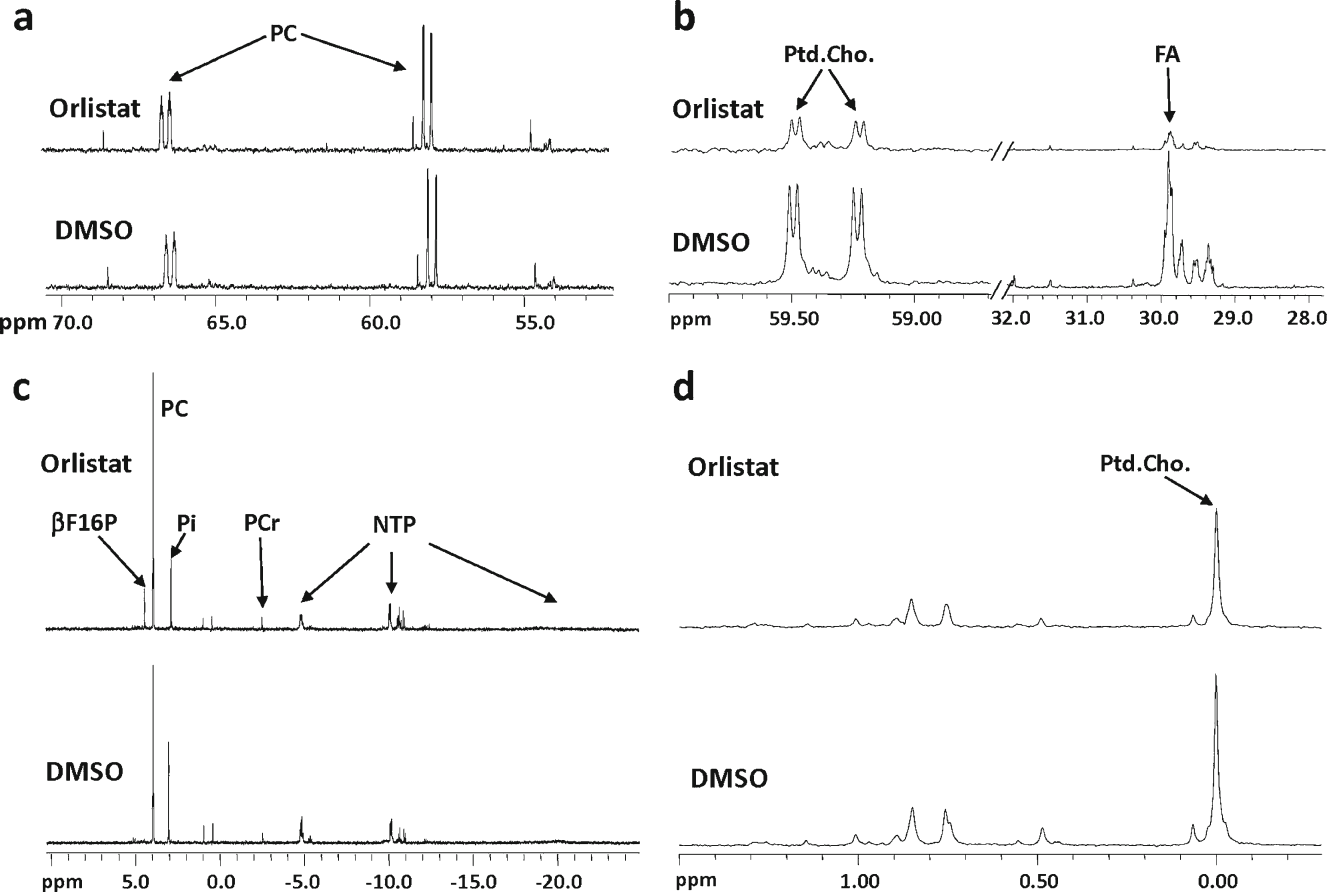
^13^C MR spectra of **a** water-soluble metabolites, **b** lipid fractions, and ^31^P MR spectra of **c** water-soluble metabolites and **d** lipid fractions of control and orlistat-treated H1975 cells incubated with [1-^13^C] d-glucose and [1,2-^13^C_2_]choline.

At baseline, in cells labeled with [1-^13^C]d-glucose and [1,2-^13^C_2_]choline, the majority of the detected signal in ^13^C MR spectra of water-soluble metabolites was observed from ^13^C-enriched metabolites, such as C3-alanine, C3-lactate, C3-glutamate, C3-glutamine, C4-glutamate, C4-glutamine, C3-aspartate, and C1 and C2-PC, α and β anomers of C1-enriched glucose (Fig. 3a). The predominant signal in ^13^C MR spectra of lipid fractions was from the methylene carbons of *de novo* FAs (Fig. 3b)*.*

When labeled with [1-^13^C]d-glucose and [1,2-^13^C_2_]choline, common metabolic differences between control and orlistat-treated H441, H1975, and PC14 cells were observed, such as increased accumulation of β-glucose (Glu-β) and β-fructose 1,6-bisphosphate (βF16P) (Fig. 3c; Fig. S2 in the “Electronic Supplementary Material”) and decreased levels of *de novo* FA and *de novo* and total phosphatidylcholine (Ptd.Cho.) However, H3255 cells did not exhibit any of the metabolic changes observed in H441, H1975, and PC14 cell lines. In orlistat-treated H3255 cells, a significant increase in the level of membrane phospholipids (CL/Ptd.EA, Ptd.serine, and sphingomyelin) was observed by ^31^P MRS, but no significant changes in *de novo* FA were observed by ^13^C MRS (Fig. S2 in the “Electronic Supplementary Material”). In contrast, orlistat-treated H441, H1975, and PC14 cells exhibited a significant decrease in membrane phospholipid levels, including Ptd.inositol (Fig. 3d; Fig. S2 in the “Electronic Supplementary Material”).

Following orlistat treatment, significantly higher total PC levels were observed by ^31^P MRS in H441 and H1975 cells; increased total choline levels were observed by ^1^H MRS in H1975 cells. However, orlistat-treated PC14 cells exhibited a decreased synthesis of *de novo* PC as compared to control cells. Additionally, increased levels of C3-lactate were observed in orlistat-treated H1975 and H3255 cells (Fig. S2 in the “Electronic Supplementary Material”).

**Fig. 4:**
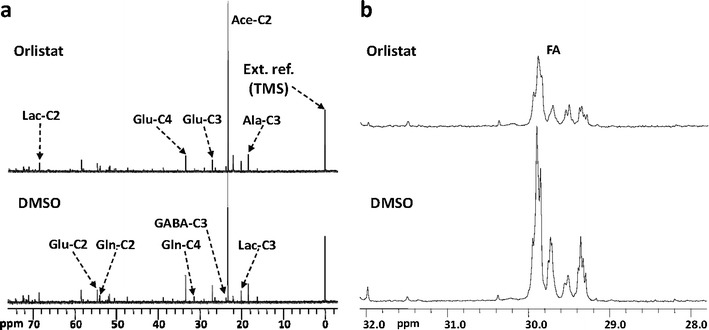
^13^C MR spectra of **a** water-soluble metabolites and **b** lipid fractions of control and orlistat-treated H1975 cells incubated with [2-^13^C]acetate.

**Fig. 5:**
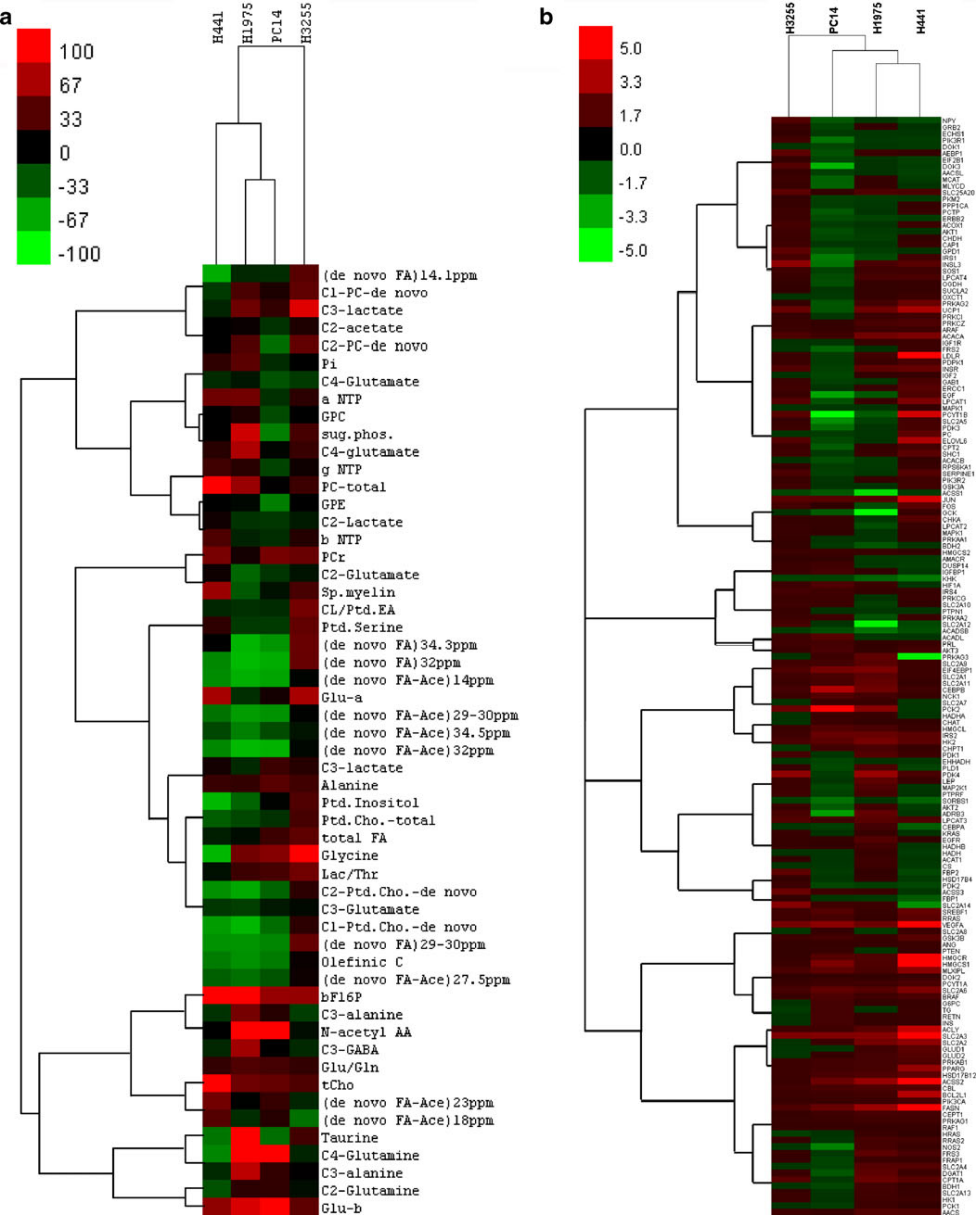
Heatmaps of **a** fold-regulation values of 168 genes in orlistat-treated NSCLC cells relative to control as determined by RT^2^-profiler PCR arrays and **b** relative changes in MRS-observed metabolite levels of orlistat-treated NSCLC cells as compared to control.

^13^C MRS with [2-^13^C]acetate labeling demonstrated that, in orlistat-treated H441 cells, C3-glutamate and taurine decreased significantly, whereas *N*-acetyl amino acids increased significantly in orlistat-treated H1975 (Fig. 4a) and PC14 cells (Fig. S2 in the “Electronic Supplementary Material”). A significant decrease in *de novo* FA synthesis was also observed following orlistat treatment in H441, H1975 (Fig. 4b), and PC14 cells, but not in H3255 cells (Fig. S2 in the “Electronic Supplementary Material”). Orlistat treatment resulted in increased labeling of C2- and C4-glutamine in PC14 cells. Fig. 5a shows the heatmap of changes in different metabolite levels as determined by ^1^H, ^13^C, and ^31^P MRS in orlistat-treated versus control NSCLC cells labeled with [1-^13^C]d-glucose and [1,2-^13^C_2_]choline or [2-^13^C]acetate. The hierarchical cluster analysis demonstrated clustering of H3255 cells farther apart from the other three cell lines. The H441 and H1975 cells were clustered much closer.

### Changes in Gene Expression Profiles After Treatment with Orlistat

Fig. 5b shows a heatmap of up^+^ or down^−^ fold-regulation of 168 genes involved in key metabolic pathways related to FA metabolism in different NSCLC cell lines treated with orlistat as compared to control. Orlistat treatment resulted in several characteristic changes in gene expression profiles, including a significant up-regulation of FASN in all the cell lines studied and up-regulation of acetyl–coenzyme A carboxylase alpha (ACACA), ATP-citrate lyase (ACLY), hexokinase-2 (HK2), low-density lipoprotein receptor (LDLR), acyl–CoA synthetase short-chain family member 2 (ACSS2), and 3-hydroxy-3-methylglutaryl-coenzyme A synthase-1 (soluble) HMGCS1 and down-regulation of acyl–CoA synthetase short-chain family member-1 (ACSS1) in majority of the NSCLC cell lines studied (Fig. S3 in the “Electronic Supplementary Material”). However, several genes were down-regulated including fructose-1,6-bisphosphatase 1 and 2 (FBP1) and (FBP2) in H441 cells. A list of genes expressing significant changes in expression levels following orlistat treatment in different NSCLC cell lines is presented in Table S5 in the “Electronic Supplementary Material”.

**Fig. 6:**
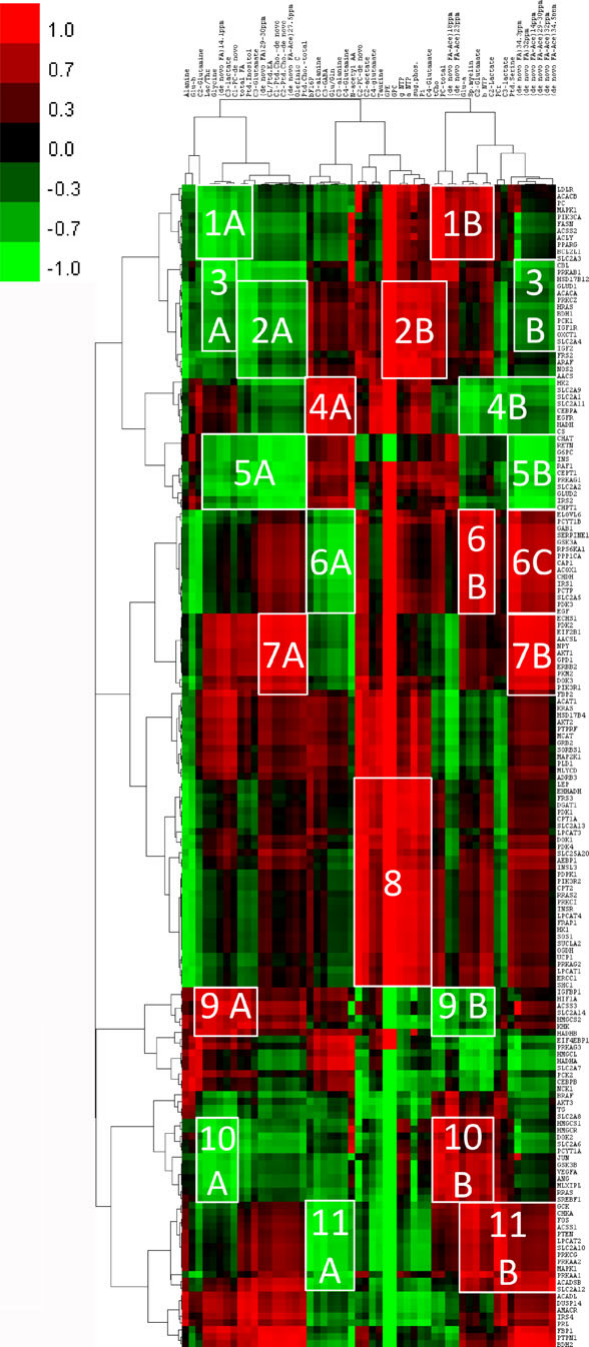
Heatmap of correlation coefficients of fold-regulation values of 168 genes and relative changes of MRS-observed metabolite levels of orlistat-treated NSCLC cells as compared to control.

The hierarchical cluster analysis of the gene expression profiles has also demonstrated that H3255 cells are clustered farther apart from the other three cell lines, followed by PC14, whereas H441 and H1975 cells were clustered together. An extensive correlation analysis was performed to find the relationships between the *in vitro* accumulation rate of different radiotracers, intermediary metabolite levels observed by MRS, and the gene expression profiles. The heatmap of gene-metabolite correlation coefficients is shown in Fig. 6. Several clusters of significantly correlating genes and metabolites were identified (Fig. S4 in the “Electronic Supplementary Material”) and marked on the heatmap (Fig. 6).

In cluster-1A, changes in concentrations of C2-glutamine, Lac/Thr, glycine, and C3-lactate, as well as *de novo* and total lipid metabolites, were inversely correlated with changes in the expression levels of genes involved in FA and lipid metabolism (FASN, ACACB, ACSS2, ACLY, LDLR) and other regulatory genes such as MAPK1, PIK3CA, and PPARG which regulate FA storage and glucose metabolism and BCL2L1 (Fig. S4-1a in the “Electronic Supplementary Material”). In cluster-1B, changes in concentrations of lipid metabolites and Glu-α, β-NTP, and amino acids (Fig. S4-1b in the “Electronic Supplementary Material”) were directly correlated with changes in expression levels of the same genes as in cluster-1A (Fig. S4-1a in the “Electronic Supplementary Material”).

Cluster-2A demonstrates the inverse correlation between changes in concentrations of *de novo* and total lipid metabolites and changes in the expression levels of genes involved in FA and lipid metabolism (ACACA, AACS), ketone metabolism (BDH1, OXCT1), insulin signaling (IGF1R, IGF2), and other genes such as GLUD1, PRKCZ, PCK1, and SLC2A4 (Fig. S4-2a in the “Electronic Supplementary Material”). Cluster-2B shows that changes in concentrations of metabolites derived from ^31^P MRS (Fig. S4-2b in the “Electronic Supplementary Material”) were directly correlated with changes in the expression levels of the same genes in cluster-2A (Fig. S4-2a in the “Electronic Supplementary Material”).

Changes in expression levels of genes, including PRKAB1 and PRKCZ encoding regulatory sub-units of AMPK—the key regulator of energy metabolism, SLC2A4 encoding GLUT-4, GLUD1, genes involved in FA and lipid metabolism (ACACA, AACS), ketone metabolism (BDH1, OXCT1), and insulin signaling (IGF1R, IGF2), were inversely correlated with changes in concentrations of metabolites in cluster-3A (Fig. S4-3a in the “Electronic Supplementary Material”) and of *de novo* FA metabolites in cluster-3B (Fig. S4-3b in the “Electronic Supplementary Material”). Changes in the expression levels of genes involved in glucose metabolism (HK2, SLC2A1, SLC2A9, SLC2A11), EGFR, and CS were directly and inversely correlated with changes in concentrations of amino acid metabolites in cluster-4A (Fig. S4-4a in the “Electronic Supplementary Material”) and that of *de novo* FA and ^31^P MRS metabolites in cluster-4B (Fig. S4-4b in the “Electronic Supplementary Material”), respectively.

Changes in concentrations of amino acids, lipid metabolites, *de novo* FA, and *de novo* Ptd.Cho. in cluster-5A (Fig. S4-5a in the “Electronic Supplementary Material”) and that of *de novo* FA metabolites in cluster-5B (Fig. S4-5b in the “Electronic Supplementary Material”) were inversely correlated with the change in expression levels of genes involved in insulin signaling (INS, IRS2), GLUD2, G6PC, PRKAG1 encoding regulatory subunit of AMPK, and SLC2A2 encoding GLUT-2. In cluster-6A, changes in concentrations of βF16P and amino acids were inversely correlated with changes in expression levels of genes involved in phospholipid metabolism (CHDH, PCYT1B, PCTP, ACOX1, ELOVL6) and other genes such as GSK3A, IRS1, PDK3, and EGF (Fig. S4-6a in the “Electronic Supplementary Material”). However, changes in concentrations of metabolites in cluster-6B and *de novo* FA metabolites in cluster-6C were directly correlated with changes in the expression levels of these genes (Fig. S4-6b, c in the “Electronic Supplementary Material”).

Fig. S4-7a, b in the “Electronic Supplementary Material” demonstrates a direct correlation between changes in concentrations of total as well as *de novo* FA and other lipid metabolites with changes in the expression levels of genes including those involved in lipid metabolism (AACSL, ECHS1), glycolysis (PIK3R1, AKT1, PDK2, GPD1, PKM2, and FBP2), and ERBB2. In cluster-8, changes in concentrations of ^31^P MRS metabolites were directly correlated with changes in the expression levels of genes involved in FA and lipid metabolism (EHHADH, DGAT1, LPCAT1, LPCAT3, LPCAT4, CPT1A, CPT2, SLC25A20), glycolysis, and tricarboxylic acid (TCA) cycle (PDK1, PDK4, SUCLA, HK1, OGDH) (Supplementary Fig. S4-8 in the “Electronic Supplementary Material”).

In cluster-9A, changes in concentrations of amino acid and lipid metabolites were directly correlated with changes in the expression levels of genes such as HIF1A, HMGCS2, KHK, and IGFBP1 (Supplementary Fig. S4-9a in the “Electronic Supplementary Material”), whereas changes in the concentrations of metabolites in cluster-9B were inversely correlated with changes in the expression levels of these genes (Fig. S4-9b in the “Electronic Supplementary Material”). Changes in the expression levels of genes including those involved in ketone metabolism (HMGCS1), cholesterol synthesis (HMGCR), glucose metabolism (SLC2A6), and lipid metabolism (SREBF1) and other genes such as VEGFA and JUN were inversely (Fig. S4-10a in the “Electronic Supplementary Material”) and directly (Fig. S4-10b in the “Electronic Supplementary Material”) correlated with changes in the concentrations of metabolites in clusters-10A and 10B, respectively. Fig. S4-11a, b in the “Electronic Supplementary Material” demonstrate that changes in the expression levels of genes involved in choline and phospholipid metabolism (CHKA, ACSS1, LPCAT2, ACADSB), AMPK regulatory sub-units (PRKAA1, PRKAA2), and glucose transport and metabolism (SLC2A10, SLC2A12, GCK) were inversely correlated with changes in the concentrations of metabolites including amino acids (alanine, Glu/Gln, glutamine, *N*-acetyl AA, GABA) and βF16P (Fig. S4-11a in the “Electronic Supplementary Material”) and directly correlated with changes in the concentrations of metabolites in cluster-11B including *de novo* FA metabolites (Fig. S4-11a in the “Electronic Supplementary Material”).

Correlation analysis further demonstrated that the accumulation rates of [^3^H]acetate (*Ki*_Ace_) and [^14^C]fluoroacetate (*Ki*_FAce_) were directly correlated with the mRNA levels of FBP1, GPD1, PKM2, PTPN1, and BDH2 and inversely correlated with ACSS2 (*r* > 0.8, *p* < 0.05). The accumulation rate of [^18^F]FDG (*Ki*_FDG_) was directly correlated with PCK2 and HIF1A, whereas it was inversely correlated with SLC2A4, DGAT1, EHHADH, CPT2, OXCT1, and BDH1 (*r* > 0.8, *p* < 0.05). Both *Ki*_Ace_ and *Ki*_FAce_ were also directly correlated with the levels of several metabolites, such as glycine, total FA, *de novo* FA, *de novo* Ptd.Cho., Ptd. inositol, and total Ptd.Cho (*r* > 0.8, *p* < 0.05).

## Discussion

Non-invasive molecular imaging techniques such as positron emission tomography (PET) and MRS can provide the means for visualization and quantification of FASN activity in tumors, monitoring of pharmacodynamic effects, and prediction of therapeutic responses in individual patients. Several previous studies reported the application of PET imaging with [^18^F]FDG or [^11^C]-acetate for monitoring tumor therapy with C75 [14, 15]. However, PET imaging has not been used for characterization of changes in tumor metabolism resulting from orlistat-induced inhibition of FASN due to uncertainty of pharmacodynamic changes in metabolic pools and signal transduction pathways that could serve as biomarkers of treatment response.

In the current study, the effect of FASN inhibition in the cellular uptake of the metabolic precursors of fatty acids was investigated for the first time in NSCLC cell lines with different molecular and genetic abnormalities that affect FASN expression activity levels by comparing the *in vitro* accumulation of [^3^H]acetate, [^14^C]fluoroacetate (as a surrogate of [^18^F]fluoroacetate), and [^18^F]FDG. In all the cell lines studied, the predominant accumulation of [^3^H]acetate and [^14^C]fluoroacetate, as compared to [^18^F]FDG, demonstrated that these cell lines have a relatively low glycolytic activity and are more dependent on *de novo* FA synthesis for their metabolic needs and membrane biosynthesis. A significant decrease in the accumulation of [^3^H]acetate and [^14^C]fluoroacetate relative to [^18^F]FDG was observed in orlistat-treated H441 and H1975 cell lines, but not in H3255 and PC14. This is most likely due to the incomplete inhibition of FASN activity in H3255 and PC14 cells by 30 μM of orlistat, as determined by FASN activity assay (Fig. 1), which is due to the compensatory up-regulation of FASN expression levels in these cell lines, as determined by RT-PCR (Fig. 5b; Fig. S3 in the “Electronic Supplementary Material”).

There are several possible reasons to the observed decrease in [^3^H]acetate and [^14^C]fluoroacetate accumulation in orlistat-treated H441 and H1975 cell lines. During cellular metabolism, acetate is activated into acetyl–CoA by acetyl–CoA synthetase (ACS). ACSS1 and ACSS2 are the two isoforms of ACS. ACSS1 is a mitochondrial enzyme that produces acetyl CoA for oxidation in the TCA cycle. Mitochondrial acetyl–CoA produced by ACSS1 becomes citrate, which is transported into the cytosol for lipid synthesis. ACSS2 is a cytosolic enzyme that converts acetate to acetyl–CoA for the synthesis of FAs and cholesterol. Other reports concluded that ACSS2 has no major role in lipid biosynthesis in glycolytically active [^18^F]FDG-avid tumor cells based on the observation of low [^11^C]acetate accumulation [16]. In contrast, both ACSS1 and ACSS2 were highly expressed in cells with low glycolytic activity, and both enzymes correlated well with [^11^C]acetate accumulation [16]. These findings confirm that ACS is one of the key enzymes in radio-labeled acetate accumulation and acetate-dependent lipid biosynthesis in tumors with a low glycolytic phenotype [16]. In the current study, in all the NSCLC cells studied, ACSS2 expression was higher than that of ACSS1, both in control and orlistat-treated cells, demonstrating the high dependency of these cell lines on *de novo* FA for their metabolic needs. The highest level of ACSS2 expression was observed in H3255 cells, which explains the highest accumulation of [^3^H]acetate and [^14^C]fluoroacetate, as compared to other cell lines. The observed down-regulation of ACSS1 following orlistat treatment explains the decreased [^3^H]acetate and [^14^C]fluoroacetate accumulation in H441 and H1975 cells. However, ACSS2 was up-regulated in orlistat-treated H441, H1975, and PC14 cells, in addition to the up-regulation of several genes in FA metabolic pathway such as ACACA (that mediates conversion of acetate to acetyl–coA), FASN, and ACLY (Table S5 in the “Electronic Supplementary Material”). We hypothesize that the observed up-regulation of several genes encoding lipogenic enzymes in orlistat-treated cells is a compensatory adaptive mechanism to meet the increased demand for *de novo* FA. However, this demand was unmet in all the NSLC cell lines studied, which manifested as a decrease in *de novo* synthesis of FA as observed by ^13^C MRS (Fig. S3 in the “Electronic Supplementary Material”).

Inhibition of FASN by orlistat caused a decrease in the accumulation of not only [^3^H]acetate and [^14^C]fluoroacetate but also of [^18^F]FDG, especially in H441 and H1975 cell lines. This observation can be explained as follows. The uptake and utilization of glucose is controlled by a multitude of factors participating in the increased aerobic glycolysis of cancers. [^18^F]FDG accumulation depends on the level of expression activity of glucose transporters (GLUTs) and subsequent phosphorylation by hexokinases (HKs) [17]. Several reports have indicated that [^18^F]FDG accumulation reflects the coordinated expression of GLUTs and activity of HKs, although uncoupling of GLUTs and HKs activities in some cancers has been described as well [18–20]. Also, it has been demonstrated that [^18^F]FDG uptake decreased in cancer cells undergoing apoptosis [21]. In the present study, PC14 cells at baseline demonstrated the highest expression of HK2 and GLUT1, which explains the significantly higher accumulation of [^18^F]FDG in these cells as compared to that in other cell lines. In orlistat-treated NSCLC cells, the rate of accumulation of [^18^F]FDG negatively correlated with GLUT4 expression, while HK1 and HK2 were significantly up-regulated in H441 and H1975 cells. Also, HK2 was up-regulated selectively in PC14 cells. The observed transcriptional up-regulation of these metabolic enzymes in orlistat-treated H441, H1975, and PC14 cells in spite of the decreased accumulation of [^18^F]FDG is part of the compensatory adaptive mechanisms of response to orlistat treatment. However, this compensatory response was insufficient to increase the glycolytic activity in orlistat-treated H441, H1975, and PC14 cells, as demonstrated by the down-regulation of FBP1 and FBP2 and the accumulation of [^13^C]glucose and the glycolytic intermediate βF16bP. These observations explain the observed decrease in glucose utilization and decreased accumulation of [^18^F]FDG in these cell lines in response to orlistat treatment. Importantly, decreased accumulation of [^18^F]FDG in orlistat-treated H441 and H1975 cells was observed despite increased mRNA levels of GLUT1, GLUT4, HK-1, HK-2, VEGF, and HIF1-a, which demonstrates that the up-regulation of transcription of genes encoding for these enzymes did not however translate into increased enzymatic activity [21].

Orlistat-treated H1975 cells exhibited the largest buildup of βF16bP (445 ± 45 %) as compared to other cell lines. This may possibly be due to the PIK3CA mutation in H1975 cells in addition to the other common mutations observed in H441 and PC14 cells (Table S1 in the “Electronic Supplementary Material”). Mutant PIK3CA activates AKT signaling, which up-regulates FASN and promotes glycolysis [22]. However, inhibition of FASN by orlistat may have possibly resulted in decreased AKT signaling and inhibition of glycolysis. Another possible reason for decreased glycolysis in H441, H1975, and PC14 cells could be the significant up-regulation of PRKAB1, PRKAG1, and PRKAG2 that encode the regulatory sub-units of AMPK. AMPK activation is reported to decrease the rate of glycolysis, either by inducing the glycolysis inhibitor TIGAR [TP53 (tumor protein 53)-induced glycolysis and apoptosis regulator] via p53 activation or by AMPK-dependent mTOR suppression [23, 24]. Thus, the up-regulation of AMPK activity may explain, at least in part, the reason for decreased glycolytic activity in orlistat-treated cells despite transcriptional up-regulation of GLUTs and HKs. The results of the current study are consistent with previous report that the knockdown of FASN suppresses genes involved in glycolysis, Krebs–TCA cycle, and oxidative phosphorylation [25].

Following orlistat treatment, all the NSCLC cell lines labeled with either [^13^C]glucose or [2-^13^C]acetate exhibited decreased levels of *de novo* FA, except for H3255. This observation is also consistent with previous reports [6, 10, 13, 26]. The extent of decrease in *de novo* FA was almost the same, whether the cells were labeled with either [1-^13^C]glucose or [2-^13^C]acetate. Orlistat-treated H3255 cells exhibited a trend (albeit not statistically significant) for increased incorporation of [^13^C]glucose-derived ^13^C label into the *de novo* synthesized FA, but with no significant change in [2-^13^C]acetate incorporation. H3255 cells did not exhibit the majority of characteristic metabolic changes associated with FASN inhibition by orlistat as observed in the other cell lines. Such difference is possibly due to an insignificant inhibition of FASN activity in H3255 cells by 30 μM of orlistat in 24 h (Fig. 1) because these cells express threefold more FASN mRNA at baseline as compared to other NSCLC cell lines studied. Interestingly, PC14 cells demonstrated most of the MRS-detectable metabolic changes observed in H441 and H1975 cell lines despite insignificant inhibition of FASN enzyme activity by orlistat treatment. These results demonstrate that H3255 cells are more resistant to FASN inhibition by orlistat and are pharmacodynamically less responsive to orlistat treatment. The results of the hierarchical clustering analysis of the gene expression profiles matched very well with the *in vitro* radiotracer accumulation and MRS experiments. Both cluster analyses demonstrated that H3255 cells were clustered farther apart from other NSCLC cell lines studied, which adds to the explanation of higher resistance to FASN inhibition and lack of pronounced compensatory responses observed in other cell lines.

The decreased levels of *de novo* and total Ptd.Cho. in orlistat-treated NSCLC cell lines observed with MRS are in agreement with previous reports, indicating decreased membrane phospholipid metabolism following FASN inhibition by orlistat [10, 13]. The increased levels of *de novo* and total PC in orlistat-treated H441 and H1975 cells may be explained, at least in part, by the compensatory up-regulation of choline kinase (CHKA) in H441 and phospholipase (PLD1) in H1975 cells (Table S5 in the “Electronic Supplementary Material”). Inhibition of FASN by orlistat and the consequent changes in metabolic pool cause several adaptive changes in cellular biochemistry, which manifest in either up- or down-regulation of expression of different metabolic enzymes (Figs. S5 and S6 in the “Electronic Supplementary Material”). Relationships between *de novo* FA and phospholipid metabolites derived from the ^13^C MRS of NSCLC cells incubated with either [1-^13^C]d-glucose or [2-^13^C]acetate with the same sub-clusters of genes involved in several signaling pathways indicate that the observed results are irrespective of the source of ^13^C enrichment (Fig. S4-5a, b and 9-7a, b in the “Electronic Supplementary Material”). The direct correlation of changes in levels of ^13^C-labeled amino acid metabolites with changes in the expression levels of HK2 and genes encoding GLUTs, SLC2A1, SLC2A9, and SLC2A11 suggests co-regulation (Fig. S4-4a in the “Electronic Supplementary Material”). However, it should be noted that these genes are inversely correlated with the *de novo* FA metabolites (Fig. S4-4b in the “Electronic Supplementary Material”; Cluster-4b) due to the compensatory up-regulation of some of these genes in response to FASN inhibition by orlistat.

Additionally, the direct correlation of the genes involved in glycolysis such as FBP2, GPD1, PDK2, and PKM2 with metabolites in clusters-7A and 7B (Fig. S4-7a, b in the “Electronic Supplementary Material”) is consistent with the inhibition of glycolysis in orlistat-treated NSCLC cells. Inhibition of glycolysis is further evidenced by the increased accumulation of glycolytic intermediate βF16P, as observed both by both ^31^P and ^13^C MRS. In Fig. S4-8 in the “Electronic Supplementary Material”, genes involved in FA, lipid, and ketone metabolism and glucose transport are grouped together in one major cluster, which clearly demonstrates that these pathways are closely related to each other and that FASN inhibition by orlistat affects multiple pathways. The inverse relationship of FASN activity with levels of expression of INS, IGF2, IGF1R, IRS1, IRS2, and IGFBP1 (Fig. S4-2a, 3a, 5a, 5b, 6a, and 9b in the “Electronic Supplementary Material”) demonstrates that *de novo* FA and lipid metabolites are feedback regulated by insulin signaling pathway as reported earlier [27]. Insulin is an important regulator of FASN which not only increases the rate of FASN gene transcription but also increases gene expression and enzymatic activity [28]. The direct correlation of *de novo* FA and lipid metabolites with the expression levels of PIK3R1 and AKT1 underlines the importance of PI3K/Akt pathway in the regulation of *de novo* FA synthesis (Fig. S4-7a, b). The direct correlation of *de novo* lipid metabolites with ERBB2 (Fig. S4-7a, b in the “Electronic Supplementary Material”) is also consistent with previous reports regarding the transcriptional regulation of FASN by EGF through ERBB2 [29].

One of the important genes involved in lipogenesis from ketone bodies in adipose tissue is AACS (acetoacetyl–CoA synthetase, EC 6.2.1.16), which catalyzes the production of acetoacetate for synthesis of cholesterol and FA [30]. The observed inverse correlation between the magnitude of changes in AACS expression with changes in concentration of metabolites in cluster-2A (Fig. S4-2a in the “Electronic Supplementary Material”) suggests that FASN inhibition by orlistat in NSCLC cells induces compensatory up-regulation of AACS to satisfy the increasing demand for FA. However, this increased demand was unmet, as evidenced by decreased levels of *de novo* FA metabolites in ^13^C MRS. Furthermore, the up-regulation of PRKAB1 (Fig. S4-3b in the “Electronic Supplementary Material”) and PRKAG1 (Fig. S4-5b in the “Electronic Supplementary Material”) encoding regulatory sub-units of AMPK observed in orlistat-treated cells demonstrates the importance of FASN and FA metabolism in the production of cellular energy sources. This observation is consistent with the previous report on activation of AMPK by inhibition of FASN by C93 in human ovarian cancer cells [31]. The inverse correlation of *de novo* FA and phospholipid metabolites with the expression level of HMGCS1, HMGCS2, HMGCR, BDH1, and OXCT1 (Fig. S4-2a, 3b, 9b, and 10a in the “Electronic Supplementary Material”) suggests that orlistat treatment in NSCLC cells activates alternative metabolic pathways for energy production such as ketone and cholesterol synthesis as a compensatory response to FASN inhibition. The inverse correlation of (*Ki*) [^3^H]acetate and (*Ki*) [^14^C]fluoroacetate with the expression level of ACSS2 also demonstrates the compensatory up-regulation of ketogenesis in response to orlistat-induced inhibition of *de novo* FA synthesis.

In summary, inhibition of FASN by orlistat induces multiple adaptive changes in FA synthetic pathway and associated metabolic pathways, including induction of ketone metabolism and glutaminolysis. Furthermore, inhibition of FASN by orlistat results in the up-regulation of AMPK which demonstrates the critical role of FA metabolism in cellular energy production, which is compensated in part by induction of alternative metabolic pathways, such as ketone metabolism. These observations expand the widely accepted concept that FA synthesis is important for cell membrane biosynthesis, especially in tumor cells with low glycolytic activity. The results of this study expanded the current knowledge of the molecular mechanisms of FASN inhibition which is essential in the development of non-invasive biomarkers for therapeutic monitoring of FASN inhibition.

## Electronic Supplementary Material

Below is the link to the electronic supplementary material.

ESM 1 (PDF 718 kb)
