# Supplementary material for: Metabolic Shifts Induced by Fatty Acid Synthase Inhibitor Orlistat in Non-small Cell Lung Carcinoma Cells Provide Novel Pharmacodynamic Biomarkers for Positron Emission Tomography and Magnetic Resonance Spectroscopy
Source: Mol Imaging Biol. 2012 Aug 11;15(2):136–47. doi: 10.1007/s11307-012-0587-6 (PMC3591534; doi:10.1007/s11307-012-0587-6)
Supplement: Supplementary file 1 — (PDF 718 kb) [file 11307_2012_587_MOESM1_ESM.pdf]

Metabolic shifts Induced by Fatty Acid Synthase Inhibitor Orlistat in Non-Small Cell Lung Carcinoma Cells Provide Novel Pharmacodynamic Biomarkers for Positron Emission Tomography and Magnetic Resonance Spectroscopy.

Molecular Imaging and Biology

Madhuri Sankaranarayanapillai[1], Nianxiang Zhang[2], Keith A. Baggerly[2], Juri G. Gelovani[1*]

[1]Department of Experimental Diagnostic Imaging, [2]Bioinformatics & Comp Biology, The University of Texas M. D. Anderson Cancer Center, Houston, TX 77030.

Corresponding author: Juri G. Gelovani, Dept. of Experimental Diagnostic Imaging, UT MD Anderson Cancer Center, 1515 Holcombe Blvd, Houston, TX, 77030, USA. Phone: 713-563-3343; E-mail: jgelovani@mdanderson.org

Supplementary Material.

Cytotoxicity assay.

The viability of cells in culture following incubation for 24h with different concentrations of Orlistat were assessed using WST-1 assay (Roche, Indianapolis, IN) according to the manufacturer's protocol. Briefly, 20 x 10^3 cells per well were seeded in 96-well plates. Cells were treated with different concentrations of Orlistat for 24 hours. Control cells were treated with vehicle DMSO. Subsequently, the cells were incubated with WST-1 reagent for 4 hours and absorbance was measured at 440 nm using SAFIRE microplate spectrometer (Tecan U.S., Research Triangle Park, NC).

FASN activity assay

Tumor cells were treated with 30 μM Orlistat or DMSO for 24 hrs; then collected by trypsinization, washed twice in PBS and stored frozen at -80°C. Cell pellets were resuspended in a lysis buffer containing 1mM ethylenediaminetetraacetic acid (EDTA), 150mM NaCl, 100μg/ml Phenylmethylsulfonyl fluoride and 50mM Tris-HCl (pH 7.5), mixed thoroughly and sonicated for 2 x 15 s on ice, centrifuged at 16,000 x g for 15 min at 0°C and stored at -80°C. Protein concentration in samples was determined using Bio-Rad DC protein assay (Bio-Rad laboratories, Hercules, CA). FASN activity was determined by measuring the malonyl-CoA and acetyl-CoA-dependent oxidation of NADPH. The reaction mixture containing 200 mM potassium phosphate buffer (pH 6.6), 1 mM Dithiothreitol (DTT), 1mM EDTA, 0.24 mM NADPH and 30 μM acetyl-CoA in 0.2ml reaction volume, was added to 96 μg of particle-free supernatant. Background NADPH oxidation was monitored at 340 nm at room temperature for 3 min using TECAN Freedom Evo robotic liquid handler equipped with the SAFIRE dual monochromator-based microplate spectrometer (Tecan U.S., Research Triangle Park, NC). After the addition of 50 μM malonyl Co-A, the reaction was assayed for an additional 15 min to determine FASN-dependent oxidation of NADPH. The reaction rate was corrected for the background rate of NADPH oxidation.

***In vitro* radiotracer accumulation studies.**

The NSCLC cells were plated in 150 mm dishes in triplicate at sub-confluent densities. The cells were treated with 30 μM of Orlistat or vehicle DMSO for 24 hrs. To initiate radiotracer accumulation, culture medium was replaced with fresh medium containing 100 μCi of [^{18}F]FDG, 0.1 μCi/ml of [^{3}H]Acetate and 0.01 μCi/ml of [^{14}C]Fluoroacetate, in the presence of Orlistat/DMSO for experimental and control groups respectively. Incubation was terminated at various time points (15, 30, 60 and 120 min.), monolayer of cells were collected by scraping, transferred into 15 ml tubes and centrifuged at 1,000 g for 2 min. A 100 μl aliquot of supernatant was transferred into pre-weighed scintillation vials and the remaining medium was removed by aspiration. The cell pellet was snap-frozen on dry ice. The frozen pellets were transferred to pre-weighed scintillation vials. The samples of media and cell pellets were weighed and thoroughly re-suspended in 0.5 ml of Soluene-350 (Perkin-Elmer). The concentration of F-18 in the culture media and cell pellets were measured using gamma spectrometer Cobra Quantum (Packard). After a complete decay of F-18 radioactivity, the Ultima Gold scintillation cocktail was added to the samples and the concentration of H-3 and C-14 was measured using a dual-energy counting protocol using Tri-Carb 3100TR scintillation counter (Packard). The ratio of radioactivity concentration in cells and culture medium ($[(dpm/g)_{cells}]/[(dpm/g)_{medium}]$) was calculated for each radionuclide in each sample, plotted against time, and the rates of unidirectional accumulation (K_i) of each compound were determined using linear regression analysis, as reported previously (13).

MRS studies.

For ^{1}H and ^{13}C MRS, tetramethylsilane (Sigma-Aldrich) was used as an external reference for chemical shift and quantification. For ^{31}P MRS of water-soluble fraction, 65 μl of 100 mM EDTA (Sigma-Aldrich) was added to each sample for chelation of metal ions, and methylene diphosphonic acid (Sigma-Aldrich) was used as an internal reference. 50 μl of 6.6 mM of trimethylphosphate was used as an internal reference for ^{31}P MRS of lipid fraction.

^{1}H and proton decoupled ^{13}C and ^{31}P MR spectra were acquired on a Avance 600 MHz spectrometer (Bruker Biospin, Germany). ^{1}H MRS was performed using a 30° flip angle, 4-second relaxation delay, and 256 scans, whereas ^{13}C MR spectra were acquired using a 30° flip angle, 3.0-second relaxation delay, and 5000 scans. ^{31}P MR spectra were acquired using a 30° flip angle, 3.6-second relaxation delay, and 5000 scans. The MR spectra were analyzed using MestReC software (Mesterlab Research, Spain). Metabolite concentrations of the control and Orlistat-treated groups were normalized to the external or internal reference and to the protein concentration in individual samples. The list of intermediary metabolites measured by ^{1}H, ^{13}C and ^{31}P MRS of cells labeled with [1-^{13}C] D-glucose and [1,2-$^{13}C_2$] choline or [2-^{13}C] acetate is presented in **Supplementary Tables 3 & 4** respectively.

Gene expression analyses.

The NSCLC cells were treated with 30 µM of Orlistat or vehicle DMSO for 24 hrs. RNA was isolated and purified using QIAGEN RNAeasy kit. RNA was reverse transcribed to cDNA using RT^2 First Strand cDNA Kit (SABiosciences, MD, USA). Custom designed RT^2 profiler PCR arrays of 168 key genes involved in different metabolic pathways related to FA synthesis and metabolism were obtained from SABiosciences (MD, USA). cDNA prepared from 2 µg of total RNA was used for PCR analysis performed using SYBR Green Master Mix (SABiosciences, MD, USA). Five different housekeeping genes, beta-2-microglobulin (B2M), hypoxanthine phosphoribosyltransferase 1 (HPRT1), ribosomal protein L13a (RPL13A), glyceraldehyde-3-phosphate dehydrogenase (GAPDH) and beta-actin (ACTB) were used as internal controls. Each PCR array was measured in triplicates for control and Orlistat groups. The fold-regulation values were calculated by $\Delta\Delta$Ct method, using the RT^2 Profiler PCR array data analysis software provided by the manufacturers (SABiosciences, MD, USA).

Supplementary figure legends

Figure S1. Changes in the accumulation of [^{3}H]Acetate, [^{14}C]Fluoroacetate and [^{18}F]FDG determined by triple label-assay format in control and Orlistat-treated NSCLC cells : (**a**) H441; (**b**) H1975; (**c**) H3255; (**d**) PC14.

Figure S2. Metabolic changes associated with FASN inhibition in Orlistat-treated NSCLC cells as observed by ^{1}H, ^{13}C and ^{31}P MRS.

Figure S3. Fold-regulation values of selected genes in Orlistat-treated NSCLC cells, as compared to control, determined by RT^{2}-profiler PCR arrays.

Figure S4. Clusters of significantly correlating genes and metabolites as marked on the heatmap of correlation coefficients shown in Figure 7.

Figure S5. Summary of changes in metabolic cascades of Orlistat-treated H441 cells, as compared to control (Part-1). In H441 cells, FASN inhibition by Orlistat resulted in up-regulation of genes (red circles) encoding several enzymes involved in multiple metabolic pathways such as TCA cycle, FA β-oxidation, ketone body metabolism, choline metabolism and mevalonate pathway. Changes in MRS observed metabolites in Orlistat-treated H441 cells are indicated by orange (increased levels) and blue (decreased levels) rectangles.

Figure S6. Summary of changes in metabolic cascades of Orlistat-treated H441 cells, as compared to control (Part-2). In H441 cells, FASN inhibition by Orlistat resulted in up-regulation (red circles) and down-regulation (blue circles) of genes encoding several enzymes involved in multiple metabolic pathways such as glycolysis, TCA cycle, glutaminolysis, and lipid metabolism. Changes in MRS observed metabolites in Orlistat-treated H441 cells are indicated by orange rectangles (increased levels).

Supplementary figure S1

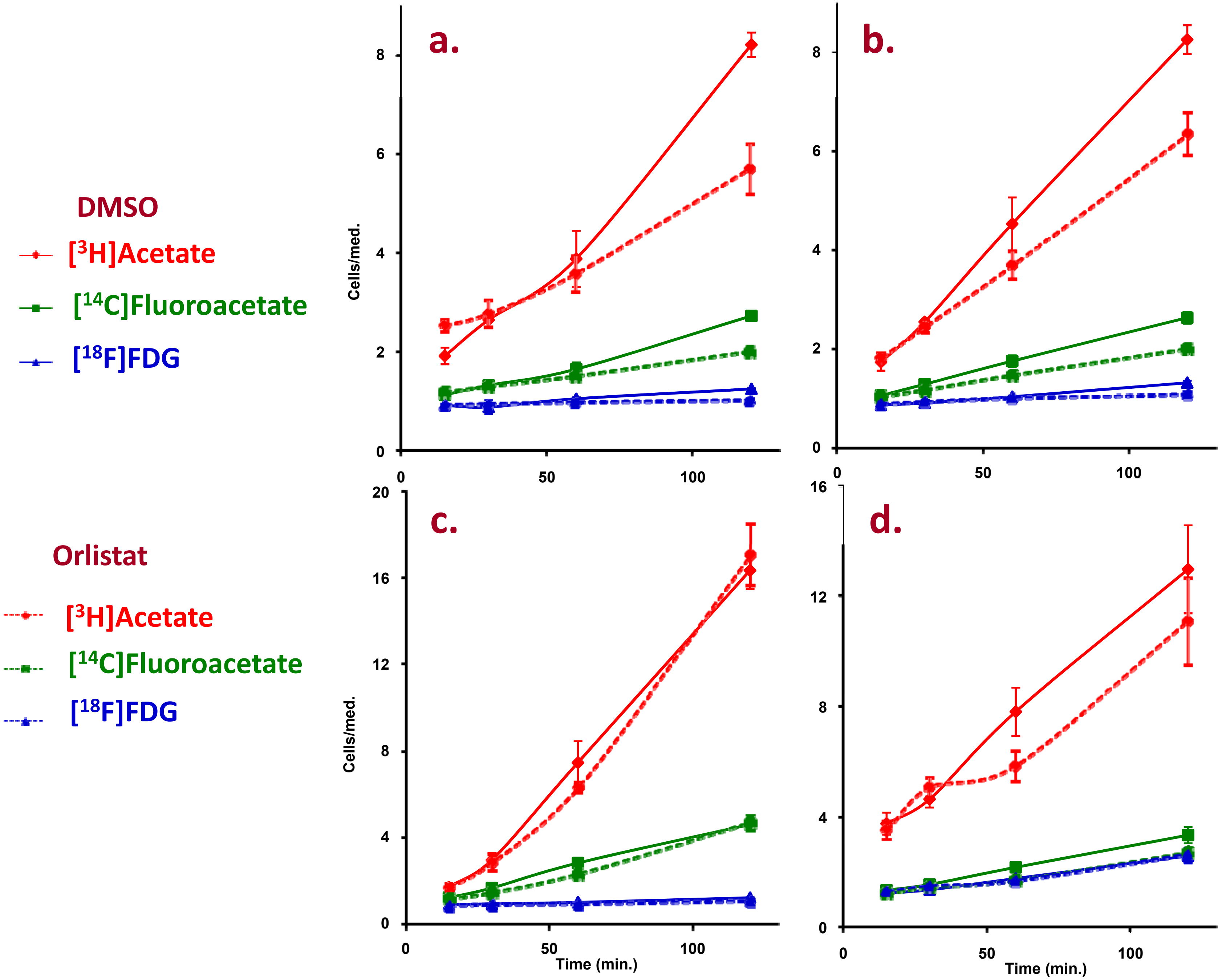

Supplementary Figure S2

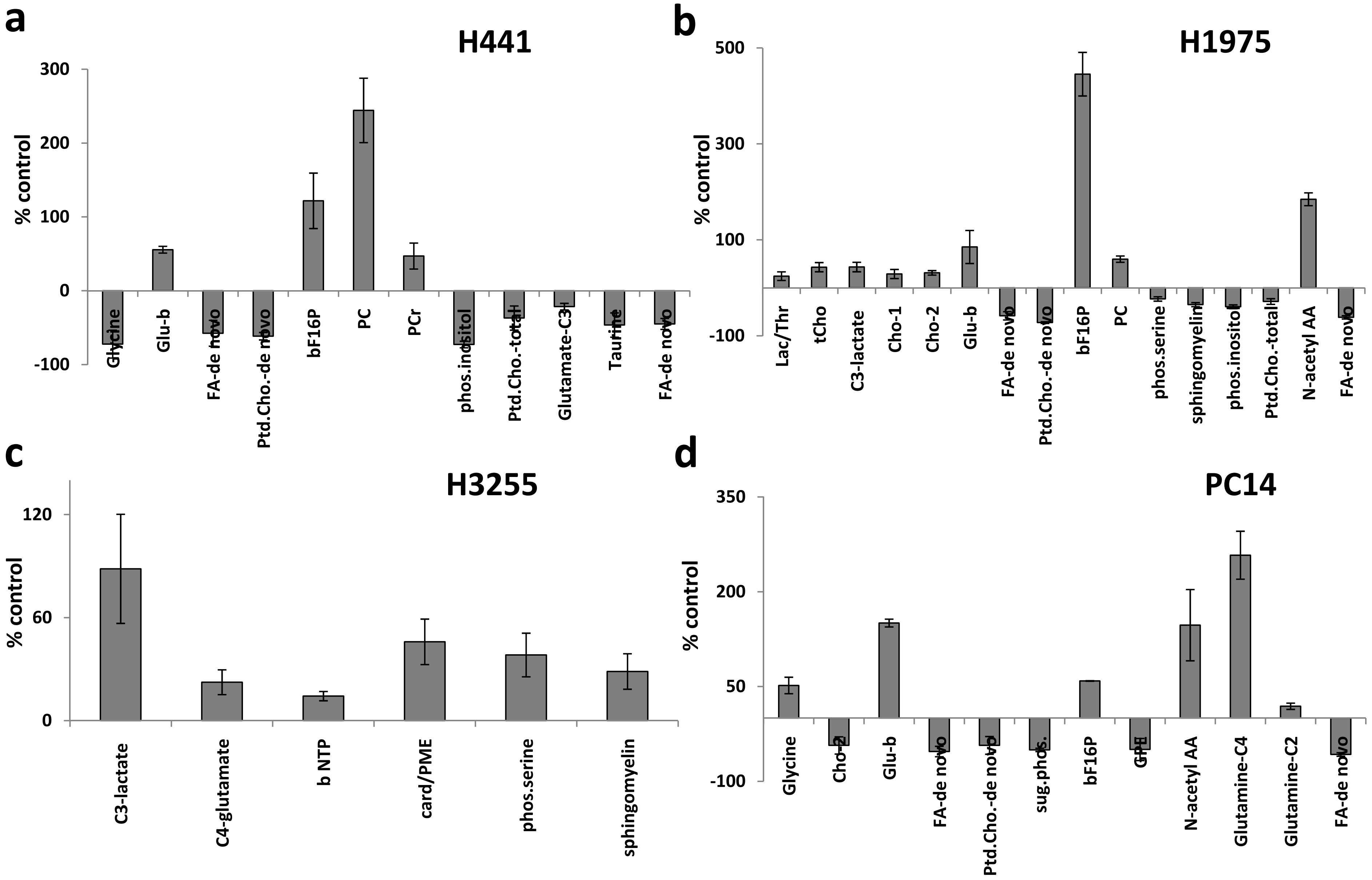

Supplementary Figure S3

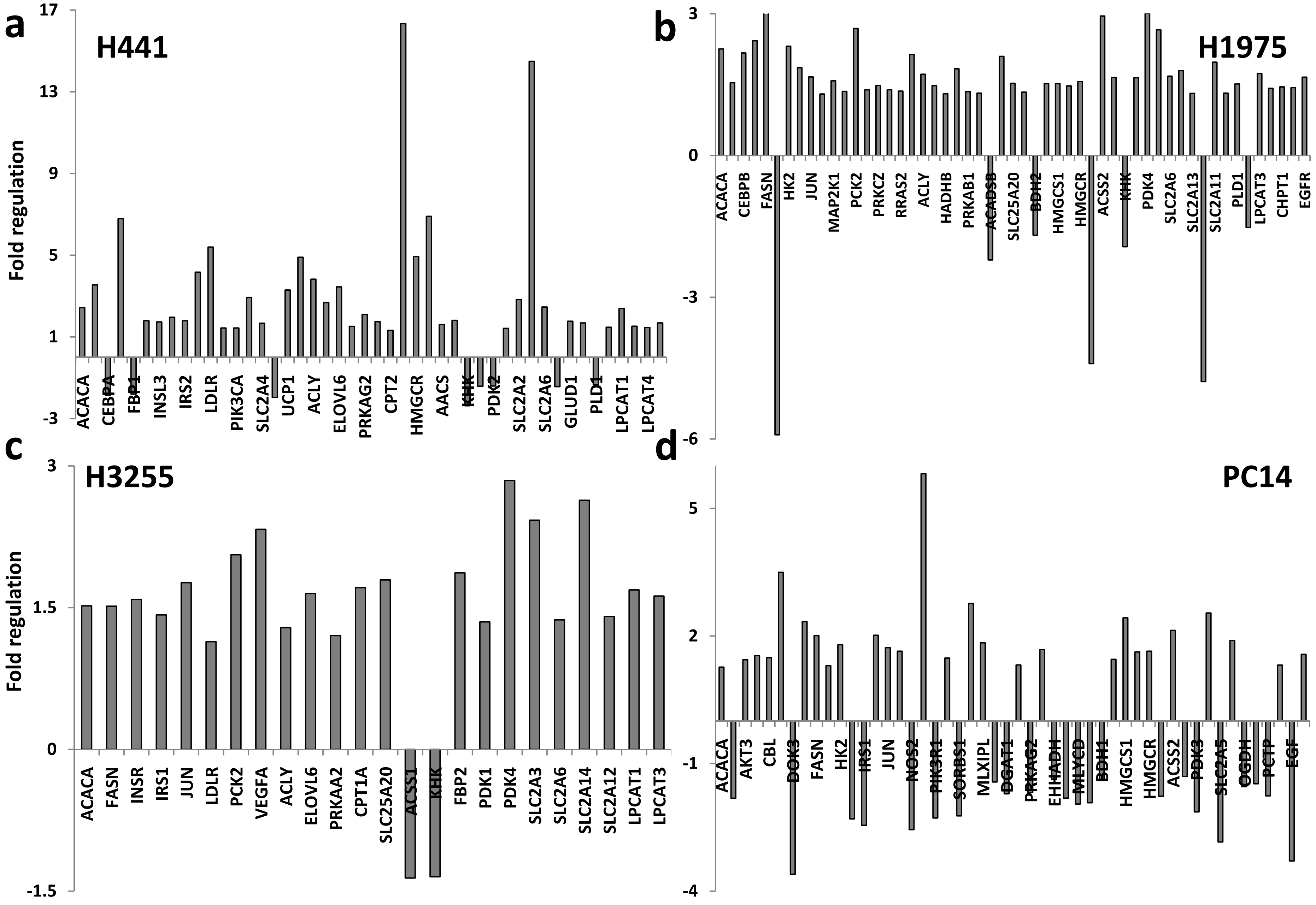

Supplementary Figure S4

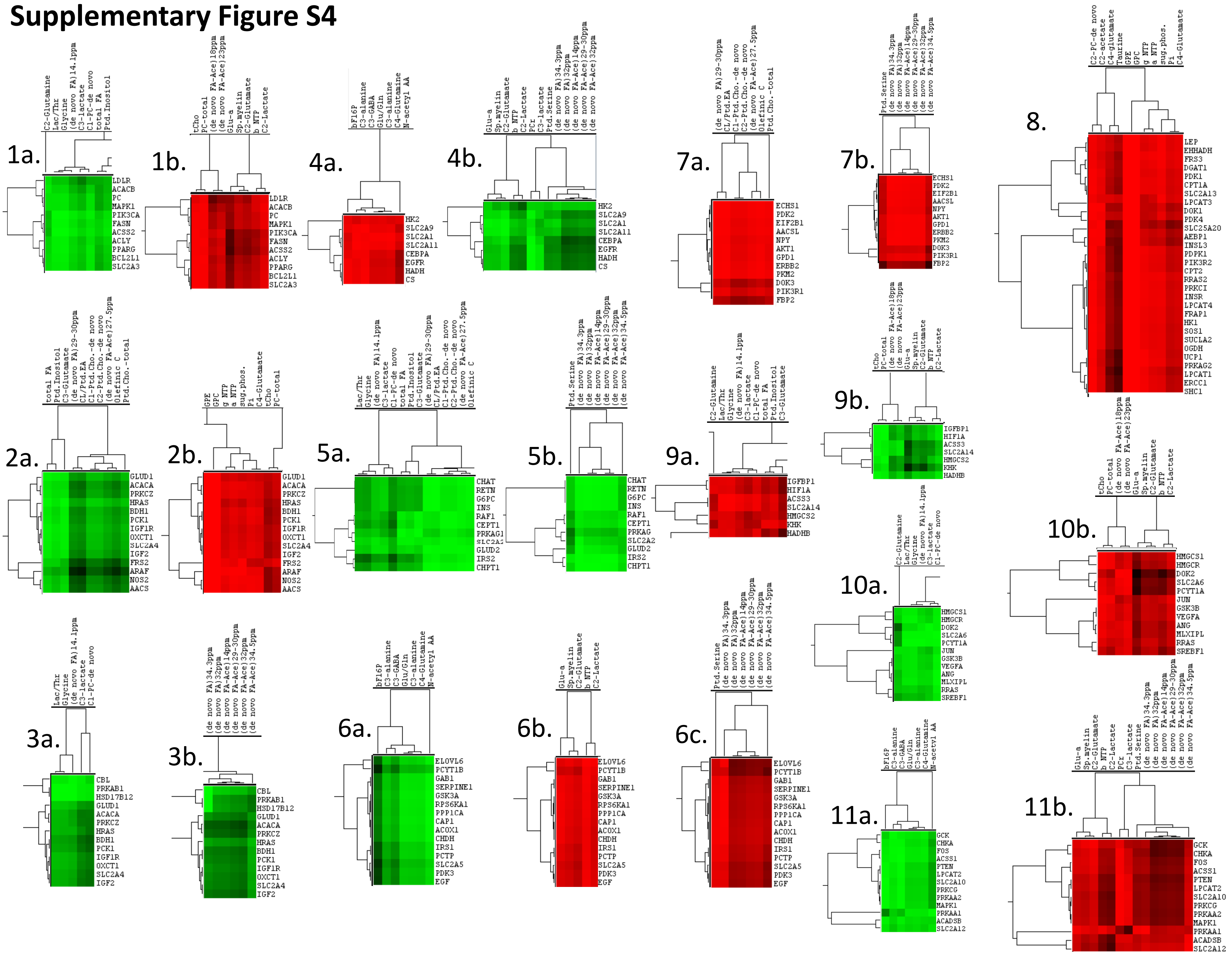

Supplementary figure S5

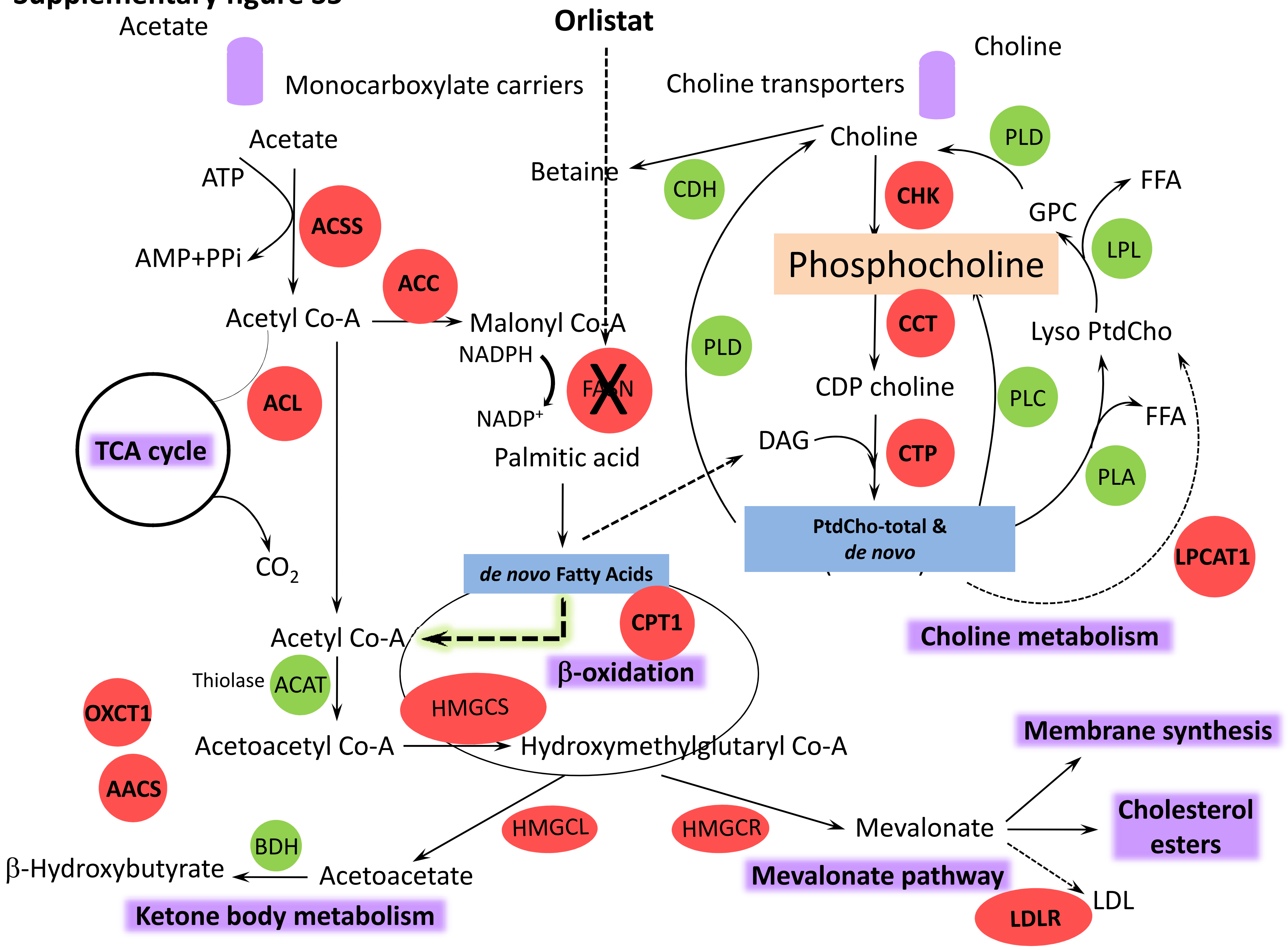

Supplementary figure S6

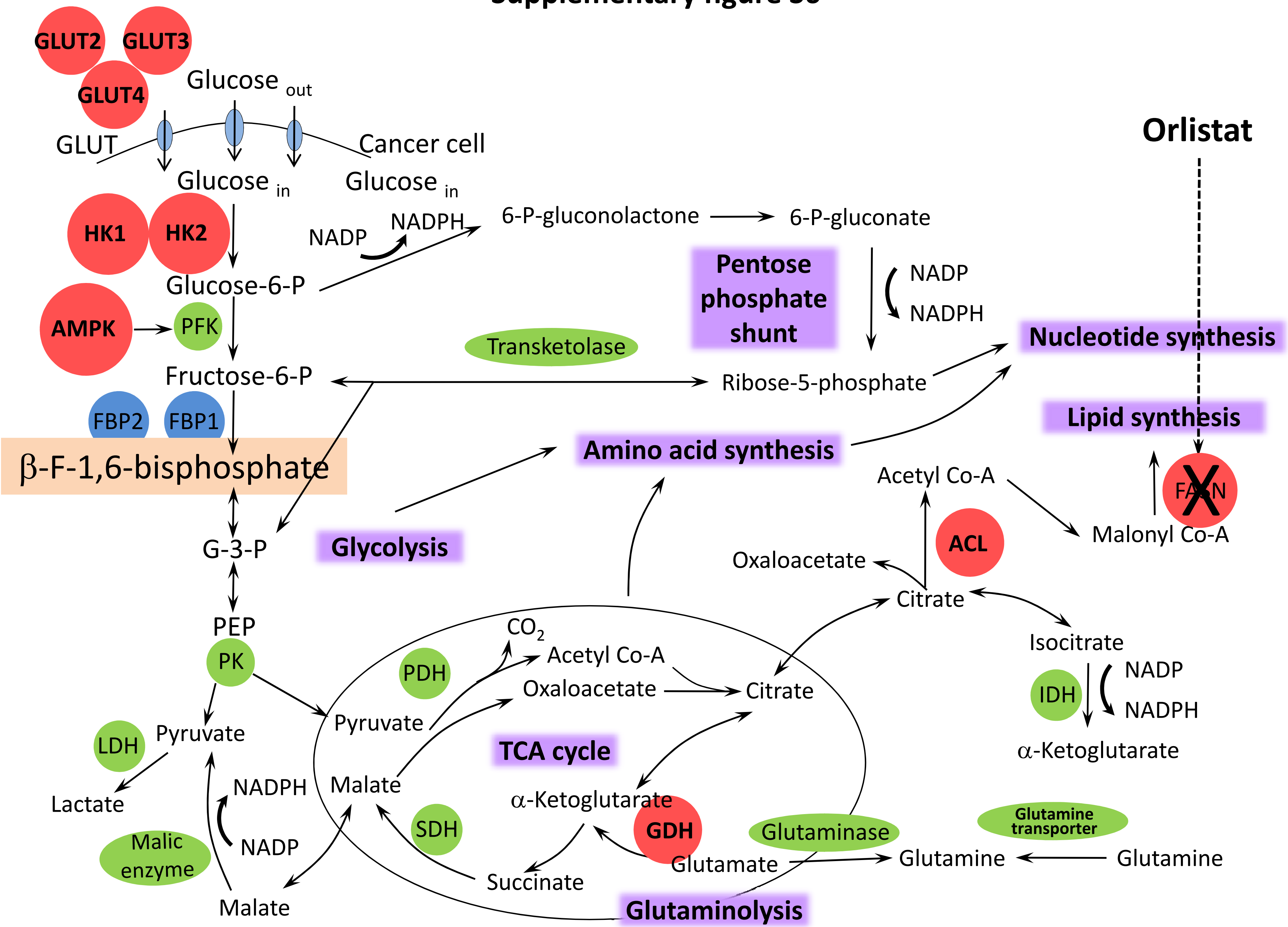

Supplementary Table 1. Genetic mutational characteristics of different NSCLC cell lines studied.

**Cell line / Mutations**	**H441**	**H1975**	**H3255**	**PC-14**
EGFR	Wild-type	Active mutant T790M & L858	Active mutant L858R	Wild-type
p53	mutant	mutant	mutant	mutant
Kras	Active mutant	Wild-type	Wild-type	Wild-type
TGF-α secretion	+++++	++	no secretion	
Other characteristics		Active mutant PIK3CA & CDKN2A	Over-expression of ErbB2 and ErbB3	Active mutant CDKN2A

Supplementary Table 2. Relative changes in the accumulation of different radiotracers determined by normalizing rate of accumulation (Ki) of [^{3}H]acetate and [^{14}C]Fluoroacetate by that of [^{18}F]FDG.

Cell line	Rate of uptake (K_i)					
	[^{3}H]Ace/[^{18}F]FDG		[^{14}C]FAce/[^{18}F]FDG		[^{3}H]Ace/[^{14}C]FAce	
	DMSO	Orlistat	DMSO	Orlistat	DMSO	Orlistat
H441	17.3 ± 1.9	33.5 ± 4.8*	4.4 ± 0.5	8.5 ± 1.3*	3.9 ± 0.1	3.9 ± 0.4
H1975	14.4 ± 0.7	23.3 ± 2.1*	3.4 ± 0.1	5.0 ± 0.4*	4.2 ± 0.1	4.7 ± 0.0*
H3255	48.2 ± 6.6	78.5 ± 18.4	10.9 ± 1.4	17.8 ± 4.1	4.4 ± 0.1	4.4 ± 0.1
PC-14	6.8 ± 0.1	5.5 ± 0.3*	1.5 ± 0.1	1.1 ± 0.0*	4.6 ± 0.2	4.9 ± 0.3

Supplementary Table 3. List of water-soluble and lipid metabolites determined by MRS of control and Orlistat-treated NSCLC cells labeled with [1-^{13}C] D-glucose & [1,2-$^{13}C_2$]choline.

MRS	Metabolite
^{1}H-aqueous	Lactate/Threonine (Lac/Thr)<br>Alanine<br>Glutamate/Glutamine (Glu/Gln)<br>Total choline (tCho)<br>Glycine
^{1}H-lipid	Total fatty acids (total FA)
^{13}C-aqueous	C_3-alanine<br>C_3-lactate<br>C_4-glutamate<br>C_1-Phosphocholine (C_1-PC-*de novo*)<br>C_2-Phosphocholine (C_2-PC-*de novo*)<br>α-Glucose (Glu-a)<br>β-Glucose (Glu-b)
^{13}C-lipid	*De novo* fatty acids (*de novo* FA)<br>*De novo* phosphatidylcholine-C_1(C1-Ptd.Cho.-*de novo*)<br>*De novo* phosphatidylcholine-C_2(C2-Ptd.Cho.-*de novo*)
^{31}P-aqueous	Sugar phosphates (Sug.phos.)<br>β-fructose 1,6-bisphosphate (bF16P)<br>Total phosphocholine (PC-total)<br>Inorganic phosphate (Pi)<br>Glycerophosphoethanolamine (GPE)<br>Glycerophosphocholine (GPC)<br>Phosphocreatine (PCr)<br>α-Nucleotide triphosphate (a-NTP)<br>β-Nucleotide triphosphate (b-NTP)<br>γ-Nucleotide triphosphate (g-NTP)

^{31}P-lipid	Cardiolipin/phosphatidylethanolamine (CL/Ptd.EA)<br>Phosphhatidylserine (Ptd.Serine)<br>Sphingomyelin (Sp.myelin)<br>Phosphatidylinositol (Ptd. Inositol)<br>Total phosphatidylcholine (Ptd.Cho.-total)

Supplementary Table 4. Fold-regulation values of genes expressing significant changes in Orlistat-treated NSCLC cells, compared to control.

MRS	Metabolite
^{13}C-aqueous	C_3-alanine<br>C_3-lactate<br>N-Acetylated amino acid (glutamate/aspartate)<br>C_2-acetate<br>C_3-gamma aminobutyric acid (C_3-GABA)<br>C_3-glutamate<br>C_4-glutamine<br>C_4-glutamate<br>Taurine<br>C_2-Glutamine<br>C_2-Glutamate<br>C_2-Lactate
^{13}C-lipid	*De novo* fatty acids (*de novo* FA-Ace)

Supplementary Table 5. Fold-regulation values of genes expressing significant changes in Orlistat-treated NSCLC cells, compared to control.

Gene name	Gene description	H441		H1975		H3255		PC-14	
		**Fold regulation**	**p value**	**Fold regulation**	**p value**	**Fold regulation**	**p value**	**Fold regulation**	**p value**
ACACA	Acetyl-Coenzyme A carboxylase alpha	2.4329	0.00147	2.2553	0.00405	1.5185	0.01357	1.2687	0.02314
ACOX1	Acyl-Coenzyme A oxidase 1, palmitoyl							-1.2058	0.00899
AEBP1	AE binding protein 1			1.1837	0.0158				
AKT2	V-akt murine thymoma viral oncogene homolog 2							-1.815	0.01181
AKT3	V-akt murine thymoma viral oncogene homolog 3 (protein kinase B, gamma)							1.4406	0.00003
ARAF	V-raf murine sarcoma 3611 viral oncogene homolog			1.2628	0.02343				
BCL2L1	BCL2-like 1	3.5455	0.00219						
BRAF	V-raf murine sarcoma viral oncogene homolog B1	1.46	0.00496	1.2311	0.01952			1.5404	0.01121
CAP1	CAP, adenylate cyclase-associated protein 1 (yeast)	1.1429	0.00922			1.2449	0.00404		
CBL	Cas-Br-M (murine) ecotropic retroviral transforming sequence	1.681	0.00022	1.5404	0.00384	1.2535	0.00360	1.488	0.00008
CEBPA	CCAAT/enhancer binding protein (C/EBP), alpha	-1.8498	0.00358						
CEBPB	CCAAT/enhancer binding protein (C/EBP), beta			2.1685	0.01273			3.4983	0.00499
DOK3	Docking protein 3							-3.605	0.00990
DUSP14	Dual specificity phosphatase 14	-1.212	0.01935					1.2628	0.04510
EIF2B1	Eukaryotic translation initiation	-1.2518	0.00584					-1.3883	0.00071

	factor 2B, subunit 1 alpha, 26kDa								
EIF4EBP 1	Eukaryotic translation initiation factor 4E binding protein 1			2.4284	0.00069			2.3403	0.00002
ERCC1	Excision repair cross-complementing rodent repair deficiency, complementation group 1 (includes overlapping antisense sequence)	1.6966	0.01005						
FASN	Fatty acid synthase	6.7865	0.00258	3.0244	0.00525	1.515	0.04012	2.0093	0.04252
FBP1	Fructose-1,6-bisphosphatase 1	-1.7992	0.01927						
FOS	V-fos FBJ murine osteosarcoma viral oncogene homolog	1.7565	0.01653						
FRAP1	FK506 binding protein 12-rapamycin associated protein 1	1.1535	0.01283	1.1947	0.02164	1.0738	0.02405	-1.3074	0.0022
FRS2	Fibroblast growth factor receptor substrate 2	1.1859	0.02662					-1.9954	0.00004
GAB1	GRB2-associated binding protein 1	1.46	0.03124			1.1914	0.01048	-1.2953	0.01124
GCK	Glucokinase (hexokinase 4)			-5.9107	0.01827				
GSK3B	Glycogen synthase kinase 3 beta	1.7443	0.00185	1.154	0.02146	1.1914	0.04029	1.3013	0.01372
HK2	Hexokinase 2	1.7892	0.01825	2.3134	0.02668			1.7942	0.02732
HRAS	V-Ha-ras Harvey rat sarcoma viral oncogene homolog					-1.246	0.00629		
IGF1R	Insulin-like growth factor 1 receptor	1.1969	0.02509						
INSL3	Insulin-like 3 (Leydig cell)	1.7323	0.00142					-2.3027	0.04406
INSR	Insulin receptor	1.9625	0.00617	1.8575	0.00066	1.5867	0.00061		
IRS1	Insulin receptor substrate 1					1.4234	0.02983	-2.4509	0.02938
IRS2	Insulin receptor substrate 2	1.7892	0.00073					2.0186	0.04835
JUN	Jun oncogene	4.1679	0.00064	1.6625	0.03625	1.7646	0.01589	1.7251	0.04495
KRAS	V-Ki-ras2 Kirsten rat sarcoma viral oncogene homolog					1.1116	0.02999		
LDLR	Low density lipoprotein receptor	5.3989	0.00001	1.3013	0.00149	1.1402	0.00023	-1.203	0.03237
MAP2K1	Mitogen-activated protein kinase kinase 1			1.5801	0.03456	1.1832	0.00529		

NCK1	NCK adaptor protein 1			1.3566	0.00249	1.1723	0.04510	1.6434	0.00003
NOS2	Nitric oxide synthase 2, inducible							-2.555	0.00446
PCK2	Phosphoenolpyruvate carboxykinase 2 (mitochondrial)			2.6882	0.02095	2.06	0.00141	5.8159	0.00050
PDPK1	3-phosphoinositide dependent protein kinase-1	1.4366	0.00031					-1.1865	0.04357
PIK3CA	Phosphoinositide-3-kinase, catalytic, alpha polypeptide	1.4333	0.00145			1.0887	0.01964		
PIK3R1	Phosphoinositide-3-kinase, regulatory subunit 1 (alpha)					1.2977	0.00326	-2.2815	0.00008
PKM2	Pyruvate kinase, muscle							-1.2454	0.00267
PPARG	Peroxisome proliferator-activated receptor gamma	2.9336	0.00007	1.3883	0.01953	1.2193	0.01223		
PRKCI	Protein kinase C, iota	1.3098	0.00642						
PRKCZ	Protein kinase C, zeta	1.5326	0.00236	1.4811	0.00880				
PTPRF	Protein tyrosine phosphatase, receptor type, F			1.3915	0.02334			-1.2924	0.03091
RRAS	Related RAS viral (r-ras) oncogene homolog	1.8058	0.00106						
RRAS2	Related RAS viral (r-ras) oncogene homolog 2	1.3159	0.00428	1.3629	0.00497				
SERPINE 1	Serpin peptidase inhibitor, clade E (nexin, plasminogen activator inhibitor type 1), member 1	1.654	0.00029					-1.2805	0.00319
SHC1	SHC (Src homology 2 domain containing) transforming protein 1	1.7769	0.00160			1.2977	0.03576		
SLC2A1	Solute carrier family 2 (facilitated glucose transporter), member 1							1.4811	0.03682
SLC2A4	Solute carrier family 2 (facilitated glucose transporter), member 4	1.6656	0.00090						
SORBS1	Sorbin and SH3 domain containing 1	-1.9643	0.02394			-1.287	0.03187	-2.2294	0.00187
SOS1	Son of sevenless homolog 1 (Drosophila)	1.2306	0.00681	1.2226	0.00696	1.2306	0.02145	-1.2687	0.01113

SREBF1	Sterol regulatory element binding transcription factor 1	1.9807	0.00643						
TG	Thyroglobulin	1.1615	0.01549						
UCP1	Uncoupling protein 1 (mitochondrial, proton carrier)	3.2928	0.03497						
VEGFA	Vascular endothelial growth factor A	4.8996	0.00010	2.1386	0.00159	2.3284	0.00206	2.7638	0.00026
ACLY	ATP citrate lyase	3.8282	0.00008	1.7187	0.00048	1.2882	0.02745		
MLXIPL	MLX interacting protein-like (Carbohydrate response element-binding protein (ChREBP))	2.682	0.00023	1.479	0.02009	1.4763	0.10138	1.8387	0.01254
HADH	Hydroxyacyl-Coenzyme A dehydrogenase	-1.1701	0.03866						
HADHA	Hydroxyacyl-Coenzyme A dehydrogenase/3-ketoacyl-Coenzyme A thiolase/enoyl-Coenzyme A hydratase (trifunctional protein), alpha subunit	-1.0521	0.03728						
HADHB	Hydroxyacyl-Coenzyme A dehydrogenase/3-ketoacyl-Coenzyme A thiolase/enoyl-Coenzyme A hydratase (trifunctional protein), beta subunit			1.3056	0.00125				
ELOVL6	ELOVL family member 6, elongation of long chain fatty acids (FEN1/Elo2, SUR4/Elo3-like, yeast)	3.4502	0.00000			1.6495	0.00049	-1.432	0.02984
DGAT1	Diacylglycerol O-acyltransferase homolog 1 (mouse)			1.8336	0.01383			-1.7108	0.02435
PRKAA2	Protein kinase, AMP-activated, alpha 2 catalytic subunit					1.2047	0.04798		
PRKAB1	Protein kinase, AMP-activated, beta 1 non-catalytic subunit	1.5157	0.00116	1.3516	0.01279			1.3183	0.04214
PRKAG1	Protein kinase, AMP-activated, gamma 1 non-catalytic subunit	1.257	0.01219	1.2611	0.03673				

PRKAG2	Protein kinase, AMP-activated, gamma 2 non-catalytic subunit	2.0994	0.00113	1.3207	0.00257			-1.6833	0.02201
PRKAG3	Protein kinase, AMP-activated, gamma 3 non-catalytic subunit	-4.8121	0.00483						
ACADSB	Acyl-Coenzyme A dehydrogenase, short/branched chain	-1.544	0.01797	-2.212	0.01148				
ACADL	Acyl-Coenzyme A dehydrogenase, long chain							1.6802	0.03722
EHHADH	Enoyl-Coenzyme A, hydratase/3-hydroxyacyl Coenzyme A dehydrogenase							-1.3705	0.03582
CPT1A	Carnitine palmitoyltransferase 1A (liver)	1.7451	0.00081	2.0965	0.00005	1.7116	0.00263		
CPT2	Carnitine palmitoyltransferase 2	1.3195	0.00799					-1.8125	0.00611
SLC25A20	Solute carrier family 25 (carnitine/acylcarnitine translocase), member 20	1.488	0.03005	1.5277	0.00017	1.7925	0.00006		
MLYCD	Malonyl-CoA decarboxylase					1.1085	0.01658	-1.9516	0.00415
MCAT	Malonyl CoA:ACP acyltransferase (mitochondrial)					1.2047	0.02101	-1.9203	0.00619
AMACR	Alpha-methylacyl-CoA racemase	-1.181	0.01976	-1.1318	0.00890				
CS	Citrate synthase	-1.1408	0.0161	1.1821	0.00011				
OXCT1	3-oxoacid CoA transferase 1	1.2086	0.00207						
BDH1	3-hydroxybutyrate dehydrogenase, type 1			1.3423	0.01109			-1.3768	0.02566
BDH2	3-hydroxybutyrate dehydrogenase, type 2			-1.688	0.00008				
HSD17B4	Hydroxysteroid (17-beta) dehydrogenase 4	-1.1701	0.00086						
HSD17B12	Hydroxysteroid (17-beta) dehydrogenase 12	1.8532	0.00011	1.5241	0.00175	1.2103	0.01174	1.4526	0.01861
HMGCS1	3-hydroxy-3-methylglutaryl-Coenzyme A synthase 1 (soluble)	16.3362	0.00001	1.5206	0.00023			2.4261	0.00188

HMGCL	3-hydroxymethyl-3-methylglutaryl-Coenzyme A lyase	1.146	0.00718	1.4722	0.00202	1.1371	0.04201	1.623	0.00148
HMGCR	3-hydroxy-3-methylglutaryl-Coenzyme A reductase	4.936	0.00001	1.5634	0.00208			1.6419	0.00857
ACSS1	Acyl-CoA synthetase short-chain family member 1			-4.4035	0.00001	-1.361	0.02430	-1.767	0.02517
ACSS2	Acyl-CoA synthetase short-chain family member 2	6.9003	0.00005	2.9513	0.00002			2.1317	0.01224
ACAT1	Acetyl-Coenzyme A acetyltransferase 1			1.2323	0.00308				
AACS	Acetoacetyl-CoA synthetase	1.6021	0.00470	1.6525	0.01529				
PC	Pyruvate carboxylase	1.8108	0.00087						
KHK	Ketohexokinase (fructokinase)	-2.3729	0.00005	-1.9301	0.00005	-1.3485	0.00148	-1.3025	0.04358
FBP2	Fructose-1,6-bisphosphatase 2	-1.4142	0.03850			1.8687	0.00458		
HK1	Hexokinase 1	1.2198	0.02820	1.2495	0.02176				
PDK1	Pyruvate dehydrogenase kinase, isozyme 1	1.1674	0.00099	1.6449	0.00703	1.3491	0.01091		
PDK2	Pyruvate dehydrogenase kinase, isozyme 2	-1.4012	0.00064	-1.2559	0.03792				
PDK3	Pyruvate dehydrogenase kinase, isozyme 3	1.4175	0.00165			1.1772	0.01655	-2.1356	0.00002
PDK4	Pyruvate dehydrogenase kinase, isozyme 4			3.0063	0.00073	2.8455	0.00008		
SLC2A2	Solute carrier family 2 (facilitated glucose transporter), member 2	2.8284	0.00795						
SLC2A3	Solute carrier family 2 (facilitated glucose transporter), member 3	14.4868	0.00006	2.6598	0.00002	2.4261	0.01500	2.5409	0.00392
SLC2A5	Solute carrier family 2 (facilitated glucose/fructose transporter), member 5			-1.2852	0.03937			-2.8442	0.00138
SLC2A6	Solute carrier family 2 (facilitated glucose transporter), member 6	2.4623	0.00019	1.6795	0.00204	1.3711	0.04862	1.8947	0.02224
SLC2A7	Solute carrier family 2 (facilitated								

	glucose transporter), member 7								
SLC2A8	Solute carrier family 2 (facilitated glucose transporter), member 8	1.192	0.00023	-1.1936	0.04979				
SLC2A9	Solute carrier family 2 (facilitated glucose transporter), member 9	1.203	0.01577	1.7958	0.03477				
SLC2A14	Solute carrier family 2 (facilitated glucose transporter), member 14					2.6366	0.00746		
SLC2A13	Solute carrier family 2 (facilitated glucose transporter), member 13	1.1096	0.03878	1.3146	0.01668				
SLC2A12	Solute carrier family 2 (facilitated glucose transporter), member 12	-1.434	0.01474	-4.7855	0.00021	1.4064	0.00143		
SLC2A11	Solute carrier family 2 (facilitated glucose transporter), member 11			1.9743	0.00053				
SLC2A10	Solute carrier family 2 (facilitated glucose transporter), member 10					1.23	0.03834		
GLUD1	Glutamate dehydrogenase 1	1.7654	0.00366	1.2409	0.03795				
GLUD2	Glutamate dehydrogenase 2	1.6818	0.00341	1.3207	0.03317				
OGDH	Oxoglutarate (alpha-ketoglutarate) dehydrogenase (lipoamide)							-1.5454	0.01485
PLD1	Phospholipase D1, phosphatidylcholine-specific	-1.3472	0.01253	1.5136	0.00725			-1.4756	0.04716
PCYT1A	Phosphate cytidylyltransferase 1, choline, alpha	1.4743	0.00080	1.2495	0.03250				
PCTP	Phosphatidylcholine transfer protein					1.1772	0.03212	-1.7589	0.04315
LPCAT1	Lysophosphatidylcholine acyltransferase 1	2.395	0.00031			1.688	0.00641		
LPCAT2	Lysophosphatidylcholine acyltransferase 2	1.1173	0.04580	-1.5248	0.00230				
LPCAT3	Lysophosphatidylcholine acyltransferase 3	1.5227	0.00057	1.7347	0.00044	1.623	0.00234		
LPCAT4	Lysophosphatidylcholine acyltransferase 4	1.4641	0.00617	1.4221	0.01225				
CHPT1	Choline phosphotransferase 1	1.1045	0.04044	1.452	0.00017			1.3152	0.00756

CHKA	Choline kinase alpha	1.6857	0.00000	-1.1163	0.03874				
CHDH	Choline dehydrogenase			-1.2472	0.01080				
CEPT1	Choline/ethanolamine phosphotransferase 1	1.3503	0.00316	1.4353	0.00340				
EGF	Epidermal growth factor (beta-urogastrone)							-3.2898	0.00544
EGFR	Epidermal growth factor receptor (erythroblastic leukemia viral (v-erb-b) oncogene homolog, avian)			1.6563	0.00019				
PTEN	Phosphatase and tensin homolog	1.154	0.00285						
HIF1A	Hypoxia inducible factor 1, alpha subunit (basic helix-loop-helix transcription factor)			1.2014	0.00131	1.2187	0.02628	1.5677	0.00341
